# *Aureobasidium pullulans* Treatment Mitigates Drought Stress in *Abies koreana* via Rhizosphere Microbiome Modulation

**DOI:** 10.3390/plants12203653

**Published:** 2023-10-23

**Authors:** Mohamed Mannaa, Gil Han, Hyejung Jung, Jungwook Park, Jin-Cheol Kim, Ae Ran Park, Young-Su Seo

**Affiliations:** 1 Department of Integrated Biological Science, Pusan National University, Busan 46241, Republic of Korea; mannaa@cu.ac.kr (M.M.); croone@pusan.ac.kr (G.H.); jhj4059@pusan.ac.kr (H.J.); 2 Department of Plant Pathology, Faculty of Agriculture, Cairo University, Giza 12613, Egypt; 3 Biotechnology Research Division, National Institute of Fisheries Science, Busan 46083, Republic of Korea; jjuwoogi@korea.kr; 4 Division of Applied Bioscience and Biotechnology, Chonnam National University, Gwangju 61186, Republic of Korea; kjinc@chonnam.ac.kr (J.-C.K.); arpark9@naver.com (A.R.P.)

**Keywords:** biostimulation, drought, Korean fir, metabarcoding

## Abstract

The Korean fir tree *Abies koreana*, an endangered species in Korea, faces threats primarily from climate change-induced stress and drought. This study proposed a sustainable method to enhance *A. koreana* drought tolerance using a black yeast-like fungus identified as *Aureobasidium pullulans* (AK10). The 16S/ITS metabarcoding analysis assessed the impact of drought and AK10 treatment on the seedlings’ rhizosphere microbiome. Results revealed a profound drought influence on the microbiome, particularly affecting fungal mycobiota. Drought-stressed seedlings exhibited elevated Agaricaceae levels, opportunistic fungi generally associated with decomposition. AK10 treatment significantly mitigated this proliferation and increased the relative abundance of beneficial fungi like *Cystofilobasidium* and *Mortierella*, known biocontrol agents and phosphate solubilizers. A notable reduction in the phytopathogenic *Fusarium* levels was observed with AK10, alongside an increase in beneficial bacteria, including *Azospirillum* and *Nitrospirillum*. Furthermore, the conducted correlation analysis shed light on microbial interrelationships within the rhizosphere, elucidating potential co-associations and antagonisms. Taken together, the isolated *A. pullulans* AK10 identified in this study serves as a potential biostimulant, enhancing the drought tolerance in *A. koreana* through beneficial alterations in the rhizosphere microbiome. This approach presents a promising strategy for the conservation of this endangered species.

## 1. Introduction

The Korean fir, *Abies koreana*, is an endemic coniferous species unique to the Republic of Korea, predominantly flourishing in subalpine regions between altitudes of 1000 to 1900 m above sea level [1]. Valued for its ornamental shape and commercial potential, especially as a desirable Christmas tree, the Korean fir has been cultivated extensively for these attributes [2]. Beyond its aesthetic appeal, this species is renowned for its phytochemicals that possess notable medical benefits, including anti-cancer activity and memory enhancement [3,4]. Despite its significance, this tree, which has been naturally present on the Korean Peninsula for millennia, has seen a worrying decline in its population over the past few decades. This quick decline led the International Union of Conservation of Nature (IUCN) to categorize it as an endangered species in 2011 [5]. According to recent studies, the average mortality rate for the Korean fir reached alarming levels in 2019 [6].

The decline in the Korean fir population over recent decades is a matter of significant concern for ecologists and conservationists. Central to this issue is the growing influence of climate change and global warming. These environmental shifts have the potential to hinder the growth of Korean fir by diminishing their photosynthetic efficiency [7,8]. Further supporting this is the finding that Korean fir from more vulnerable environments show heightened expression of genes associated with heat stress, in contrast to those from more stable habitats [9]. However, temperature rise is not the only cause of such decline. The broader consequences of climate change introduce a multitude of challenges for the Korean fir population. These include heightened typhoon intensities, fluctuating soil moisture content, and changing precipitation patterns [5,10]. Drought, in particular, is emerging as an especially acute stress, endangering tree survival [11]. As rainfall patterns shift and temperatures climb, extended drought periods may intensify the existing challenges faced by these trees. The situation is further complicated by the unique geographical setting of the Korean fir habitat. Situated atop the volcanic terrain of Mt. Halla, these trees confront the difficulties presented by the relatively shallow soils, which could limit the stability offered by their restricted root systems [12]. Therefore, efforts are being made to find a sustainable approach to protect the endangered tree and enhance its resilience to environmental stresses, primarily drought.

The rhizosphere, a critical interface between plant roots and the surrounding soil, is increasingly recognized as an indispensable player in the overall health of plants [13]. The dynamic community of microbes residing in this region, termed the rhizosphere microbiome, confers a myriad of benefits to the plant host, from aiding nutrient uptake to mediating stress tolerance [14]. Previous studies have confirmed the roles of the rhizosphere microbiome role in supporting plant growth under various environmental stresses, including drought [15,16]. Drought stress profoundly influences the structure and function of rhizosphere microbial communities. Compared to the more buffered bulk soil environment, the rhizosphere microbial inhabitants confront direct implications of water scarcity, as well as the physiological shifts in their plant hosts reflected through root exudates [14]. The microbial adaptability to drought, combined with the ability to modulate plant stress responses—like the mitigation of ethylene production via ACC deaminase activity—highlights the potential of the rhizosphere as a focal point in sustainable agriculture and conservation [17,18]. Regarding the Korean fir, Han et al. [19] studied the rhizosphere microbiome structure and reported distinct microbial assemblages between healthy and declining trees. Healthy trees harbored a richer and more diverse microbiome, teeming with beneficial microbial taxa like *Bradyrhizobium* and *Rhizobium*, known for their roles in alleviating abiotic stresses. In contrast, standing dead trees presented a clear dysbiosis, with significantly reduced microbial diversity [19]. These observations suggest that an integrated understanding of the rhizosphere microbiome, especially in the context of drought stress, could offer innovative avenues to enhance the tolerance of the Korean fir by harnessing these microbial allies.

Several studies have highlighted the efficacy of microbial treatments, particularly involving diverse bioagents such as the plant growth-promoting rhizobacteria and arbuscular mycorrhizal fungi in the promotion of plant growth and alleviating drought stress in various plant species [20,21,22]. This suggests that employing biostimulants may offer a promising alternative for combatting drought stress. Therefore, this study aimed to isolate and identify a microbial agent capable of alleviating drought stress in *A. koreana*. Additionally, we sought to assess the influence of such bioagents on the composition of the rhizosphere bacterial and fungal microbiome, and to explore the intricate interactions within this microbial community. Proposing an effective biostimulant to enhance drought resilience is an important step in the broader efforts to protect this endangered tree species. Moreover, the understanding of the interactions between the rhizosphere bacterial and fungal components can offer invaluable insights, expanding our comprehension of the complex ecosystem of the tree.

## 2. Results

### 2.1. Treatment with AK10 Enhances Drought Stress Tolerance in A. koreana Seedlings

**Figure 1:**
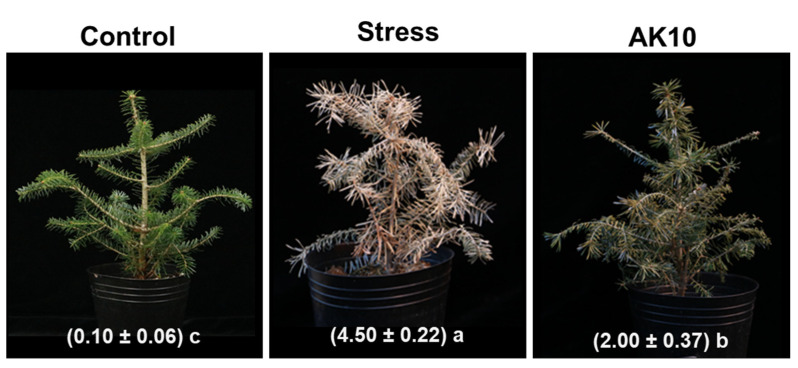
Effect of *Aureobasidium pullulans* AK10 foliar spray treatment on drought stress in *Abies koreana* seedlings. Drought stress indices were determined using a predefined scale 3 weeks post-treatment. Values below each photograph represent the mean ± standard deviation based on 3 replicates. Different lowercase letters following the indices denote statistically significant differences at *p* < 0.05, as determined by the least significant difference test. Control represents *A. koreana* seedlings under regular watering; stressed represents seedlings exposed to drought stress; and AK10 represents seedlings both exposed to drought stress and treated with *A. pullulans* AK10.

From the conducted seedling assays, seedlings exposed to drought stress exhibited pronounced needle browning and significant wilting of the shoots. However, the drought-impacted seedlings treated with AK10 through foliar spraying demonstrated improved drought tolerance. The symptoms of needle browning and wilting were markedly reduced compared to the untreated drought-stressed seedlings (Figure 1). Control seedlings that received regular watering displayed healthy growth, with vibrant green needles and no signs of wilting. This observation was verified by the drought stress index, which revealed a pronounced impact of drought stress on the affected seedlings. Notably, there was a significant (*p* < 0.05) reduction (approximately 55.56%) in these symptoms among the AK10-treated seedlings (Figure 1). This suggests that foliar treatment with AK10 enhances the drought resilience of *A. koreana*.

### 2.2. Identification of AK10 Strain

After confirmation of the role of AK10 in enhancing drought tolerance in *A. koreana* seedlings, molecular analyses of the ITS region and the *β*-tubulin gene were conducted to identify the AK10 strain. The ITS sequence analysis of AK10 demonstrated similarity levels of 99.38%, 98.53%, 98.47%, 98.43%, and 96.17% with *Aureobasidium pullulans*, *A. namibiae*, *A. leucospermi*, *A. lini*, and *A. melanogenum*, respectively. Consequently, the *β*-tubulin gene was further analyzed and revealed similarity levels of 99.05% with *A. pullulans*, 92.41% with *A. lini*, and 84.83% with *A. namibiae*.

**Figure 2:**
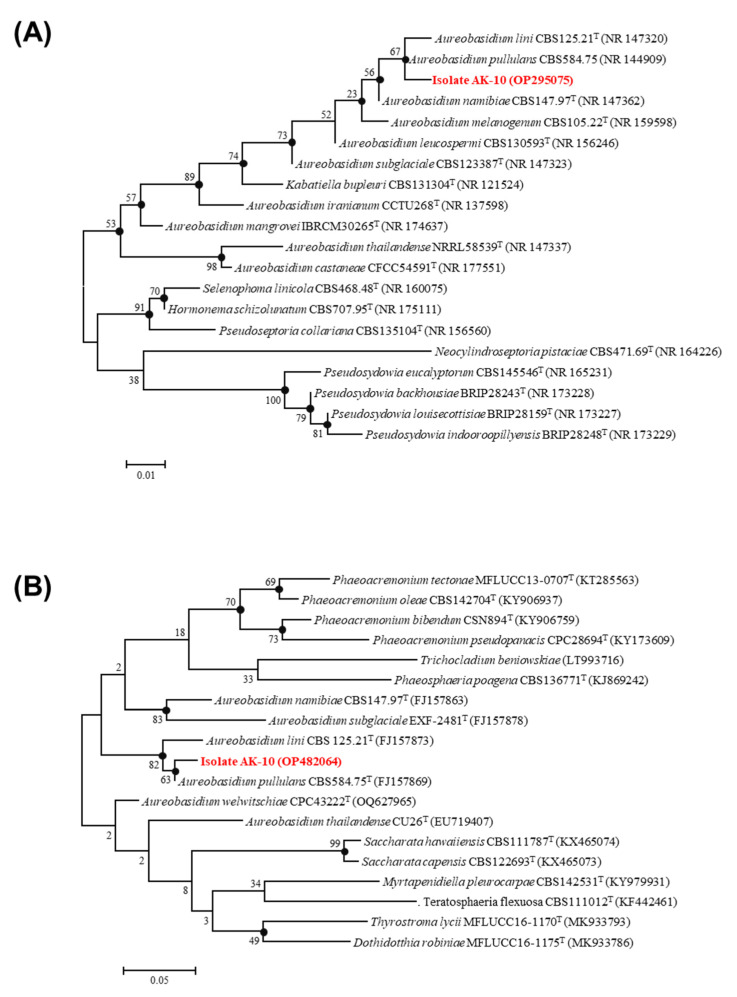
Phylogenetic relationships of isolate AK10 with related species, as determined through maximum-likelihood analysis. Trees were derived from (**A**) the ITS1-5.8S-ITS2 region and (**B**) the *β*-tubulin gene. Bootstrap values from 1000 replicates are indicated at the nodes. Nodes marked with black dots had corresponding bootstrap values exceeding 50% in trees derived using the neighbor-joining method. The scale bars denote nucleotide substitutions for every 10 or 50 nucleotide sequence lengths. Sequences are followed by the GenBank accession numbers in parentheses, with (T) denoting sequences sourced from type materials.

Phylogenetic analyses employing maximum likelihood and neighbor-joining methods for the ITS sequence failed to distinctly categorize AK10 into a unique cluster, making it challenging to differentiate AK10 specifically between *A. pullulans* and *A. lini* (Figure 2A). However, the phylogenetic tree derived from the *β*-tubulin sequence distinctly placed AK10 in a separate cluster alongside *A. pullulans* (Figure 2B). Thus, based on these results, AK10 was identified as a strain of *A. pullulans*. The sequences of both the ITS and *β*-tubulin have been deposited in the NCBI GenBank with accession numbers OP295075 and OP482064, respectively.

### 2.3. Metagenomic Analysis of 16S rRNA and ITS Sequences

High-throughput metagenomic sequencing targeting the 16S rRNA region from nine rhizosphere samples of Korean fir seedlings yielded a total of 891,220 reads, averaging 99,024 reads per sample. In contrast, sequencing of the ITS region resulted in a more substantial yield of 2,136,488 total reads, with an average count of 237,388 reads per sample. Comprehensive details of the raw sequence data for each sample, encompassing metrics such as the total base count, number of reads, GC content, Q20, and Q30 percentages, are shown in Table S1.

After sequence pre-processing, which involved merging sequences, clustering, and removing adaptors, primers, low-quality reads, and chimeras, the 16S rRNA dataset was refined to 162,218 clean non-chimeric reads. These clean reads distributed across samples had an average count of 18,024, ranging from a minimum of 15,229 to a maximum of 21,478 reads per sample.

For the ITS dataset, the final count of clean non-chimeric total reads stood at 315,523, with an average of 35,058 reads per sample, ranging from a minimum of 16,748 to a maximum of 60,933 reads. Notably, a significant portion of the identified ASVs derived from the ITS region did not belong to the Fungi kingdom. As a result, these were excluded from subsequent analyses.

### 2.4. Diversity and Richness among the Rhizosphere Microbiome

The sequencing depth was ascertained through the rarefaction curves for both the 16S rRNA and ITS sequences-derived ASVs. The 16S rRNA sequences demonstrated a satisfactory sequencing depth that could represent the primary constituents of the rhizosphere microbiome. The ASV rarefaction curve plateaued around approximately 3000 reads, confirming comprehensive representation within this depth (Figure S1A). Conversely, the rarefaction curve for the ITS sequences showed some variability. While certain samples reached a plateau, indicating a satisfactory depth, others appeared not to level off. Such results may be attributed to the presence of ITS sequences in kingdoms other than fungi, influencing the sequencing depth and ASVs representation (Figure S1B).

In assessing alpha diversity, which measures the diversity within individual samples for the bacterial microbiota, the 16S rRNA sequences revealed no statistically significant differences among the treatments. Specifically, the number of ASVs, Chao1, Shannon, and Gini–Simpson indices were comparable between the control seedlings (regularly watered) and both the drought-stressed and AK10-treated drought-stressed seedlings. In contrast, when evaluating the fungal community using ITS sequences, marked differences were evident. The drought-stressed seedlings, both untreated and treated with AK10, displayed significantly lower ASVs counts, Chao1, Shannon, and Gini–Simpson indices compared to the regularly watered control seedlings. This suggests that while drought conditions impacted the richness and diversity of the fungal community, the bacterial community remained largely undisturbed in both drought-stressed and AK10-treated drought-stressed seedlings (Table 1).

**Figure 3:**
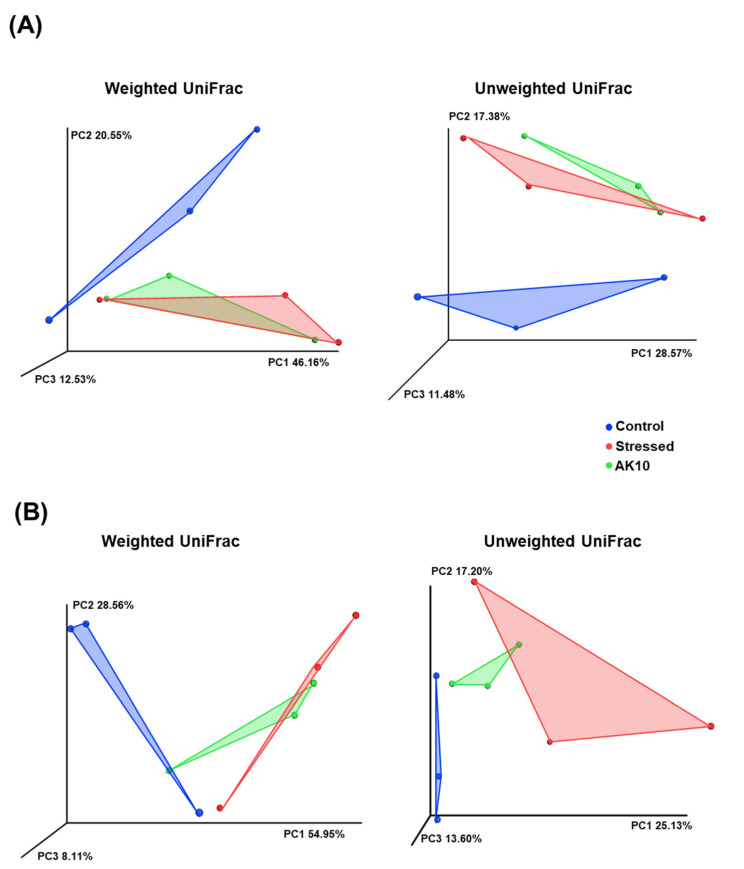
Principal coordinate analysis (PCoA) of weighted and unweighted UniFrac distances derived from (**A**) rhizosphere microbiome of *Abies koreana* seedlings using metabarcoding of the partial 16S rRNA sequence and (**B**) rhizosphere fungal community using metabarcoding of the partial ITS region sequence. Control represents *A. koreana* seedlings under regular watering; stressed represents seedlings exposed to drought stress; and AK10 represents seedlings both exposed to drought stress and treated with *Aureobasidium pullulans* AK10.

Considering beta-diversity based on 16S rRNA sequences of the rhizosphere microbiota, the PCoA plots derived from both weighted and unweighted UniFrac distances (Figure 3A) revealed distinct patterns. Control seedlings (regularly watered) clustered separately from both the drought-affected seedlings and the drought-affected seedlings treated with AK10. Notably, there was no differentiation between the AK10-treated drought-affected seedlings and their untreated counterparts. This observation suggests that drought exerted a more pronounced influence on the microbiota diversity than the AK10 treatment.

For the fungal community, as characterized by ITS sequences, control seedlings displayed a clear separation in the PCoA plot (Figure 3B). However, a slight clustering was observed between the drought-stressed seedlings, both treated and untreated with AK10, indicating subtle differences in fungal communities associated with the treatment.

### 2.5. Taxonomic Characterization and the Effects of Drought and AK10 Treatment on the Rhizosphere Microbiome Composition

**Figure 4:**
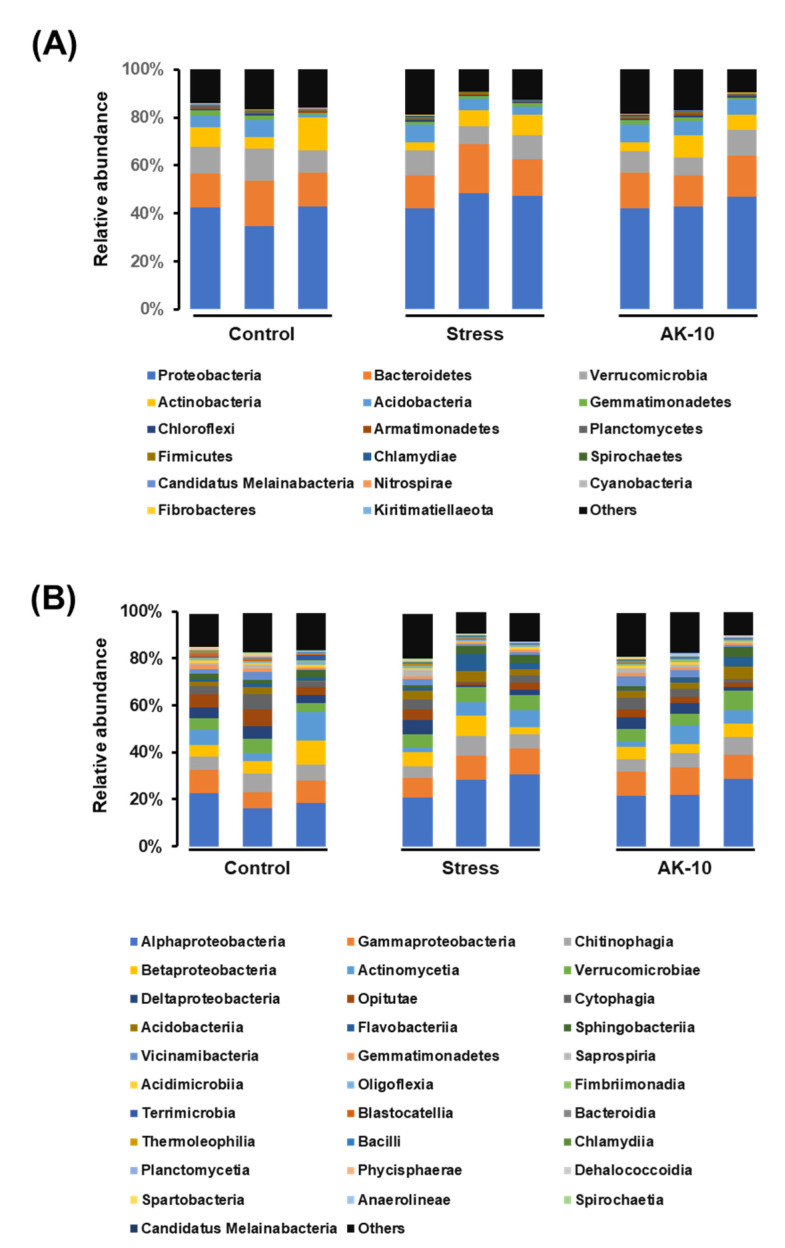
Stacked bar graphs of the microbiome structure in the rhizospheres of Korean fir seedlings, as determined by metabarcoding of the 16S rRNA region (**A**) at the class level and (**B**) at the family level. Control represents *A. koreana* seedlings under regular watering; stressed represents seedlings exposed to drought stress; and AK10 represents seedlings both exposed to drought stress and treated with *Aureobasidium pullulans* AK10.

The stacked bar graphs in Figure 4 present the relative abundances of rhizosphere microbial composition at both class and family levels in Korean fir seedlings across different treatment conditions: Regularly watered (control), drought-affected, and drought-affected seedlings treated with *A. pullulans* AK10. Predominantly, the rhizosphere microbial community of *A. koreana* at the family level was characterized by Alphaproteobacteria, Gammaproteobacteria, Chitinophagia, Betaproteobacteria, and Actinomycetia. These classes together accounted for 44.65% in the control, 79.20% in the drought, and 51.26% in the AK10 seedlings.

**Figure 5:**
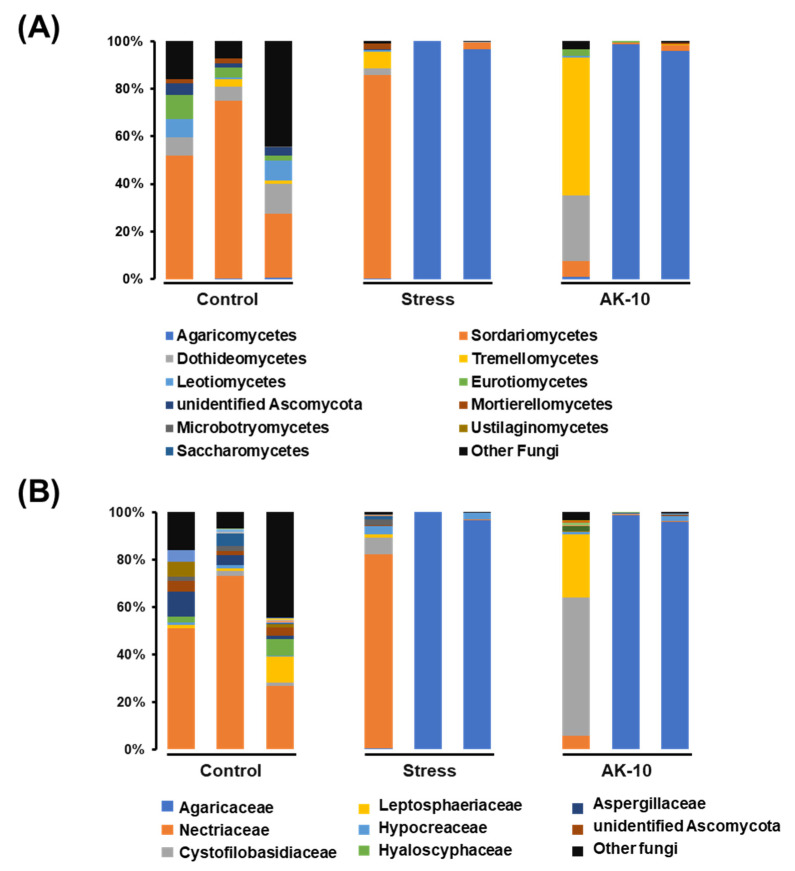
Stacked bar graphs of the fungal community structure in the rhizospheres of Korean fir, as determined by metabarcoding of the ITS region (**A**) at the class level and (**B**) at the family level. Control represents *A. koreana* seedlings under regular watering; stressed represents seedlings exposed to drought stress; and AK10 represents seedlings both exposed to drought stress and treated with *Aureobasidium pullulans* AK10.

Similarly, the stacked bar graphs in Figure 5 present the relative abundances of the rhizosphere fungal community at class and family levels under the aforementioned conditions. A notable difference was observed in the composition structure, especially pertaining to the class Agaricomycetes. Drought-affected seedlings exhibited a relative abundance of 54.38% of Agaricomycetes. This value was significantly tempered to 33.90% in seedlings that underwent drought stress but were also treated with *A. pullulans* AK10. In contrast, control seedlings that were consistently watered displayed a minimal 0.04% abundance of this class. These findings underscore that drought stress predominantly influenced this group of fungi, and the introduction of AK10 treatment managed to mitigate this impact to an extent.

The heatmap in Figure S2, based on hierarchical clustering and utilizing Euclidean distance and average linkage, shows the top 50 most abundant bacterial genera in the rhizosphere of Korean fir seedlings. While distinctions between sample clusters were limited, a slight grouping pattern was evident among drought-affected seedlings, whether treated with AK10 or not.

Conversely, Figure S3, highlighting the top 25 most abundant fungal genera, exhibited more defined clustering patterns. The entire control group distinctly clustered into one group. This prominent differentiation was largely attributed to variations in the abundances of certain genera, specifically unidentified members of Agaricaceae and Nectriaceae families, as well as *Fusarium* and *Leptosphaeria*.

### 2.6. Variations in Rhizosphere Microbial Genera of Drought-Affected A. koreana Seedlings with AK10 Treatment

**Figure 6:**
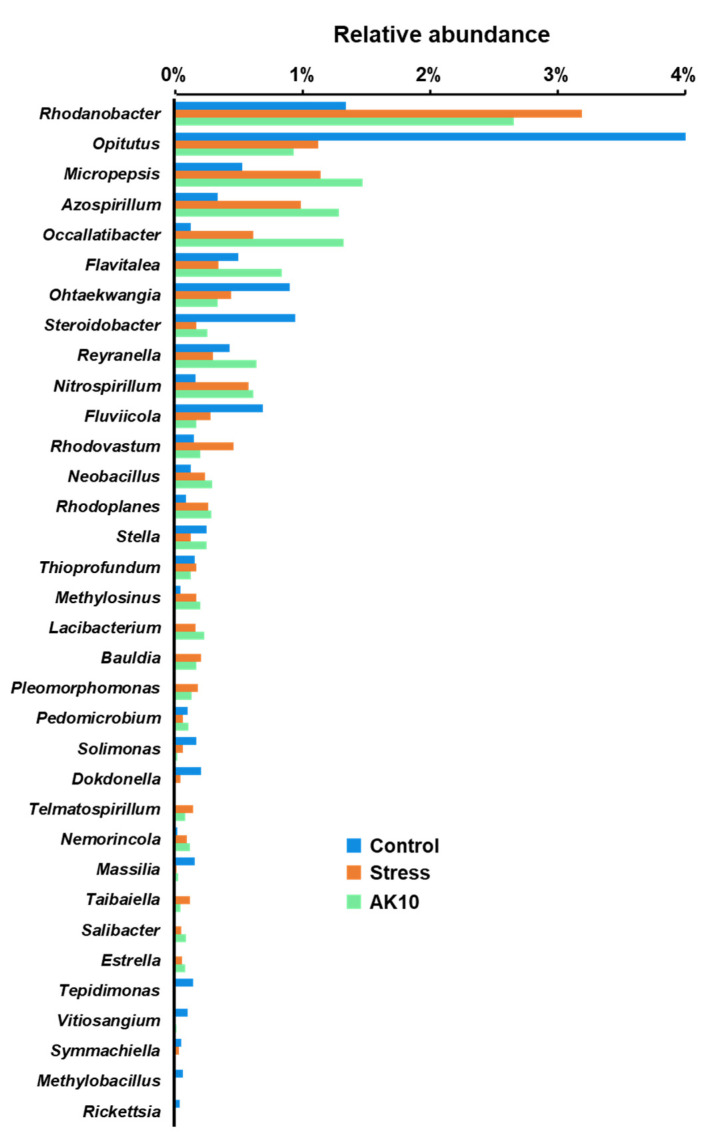
Relative abundances of bacterial genera with significant differences (*p* < 0.05) across control, stressed, and AK10-treated Korean fir seedling rhizospheres. Bars denote mean values (*n* = 3) of each bacterial genus’s relative abundance. Control represents *A. koreana* seedlings under regular watering; stressed represents seedlings exposed to drought stress; and AK10 represents seedlings both exposed to drought stress and treated with *Aureobasidium pullulans* AK10.

Building upon the observed distinctions in rhizosphere microbiota and mycobiota among control, drought-affected, and AK10-treated *A. koreana* seedlings, multiple bacterial genera were detected with significant differences in their relative abundance levels (*p* < 0.05) across the sample groups. Figure 6 presents bar graphs illustrating these differences in bacterial populations. Although the dominant bacterial taxa remained relatively stable, other bacterial taxa with low relative abundance exhibited variations. Notably, the control group exhibited higher relative abundances of genera such as *Opitutus*, *Ohtaekwangia*, *Steroidobacter*, *Stella*, *Solimonas*, *Dokdonella*, *Massilia*, *Tepidimonas*, *Vitiosangium*, *Symmachiella*, *Methylobacillus*, and *Rickettsia*. Conversely, AK10-treated seedlings displayed elevated relative abundances for genera like *Micropepsis*, *Azospirillum*, *Occallatibacter*, *Flavitalea*, *Reyranella*, *Nitrospirillum*, *Neobacillus*, *Rhodoplanes*, *Methylosinus*, *Lacibacterium*, *Nemorincola*, *Salibacter*, and *Estrella*.

**Figure 7:**
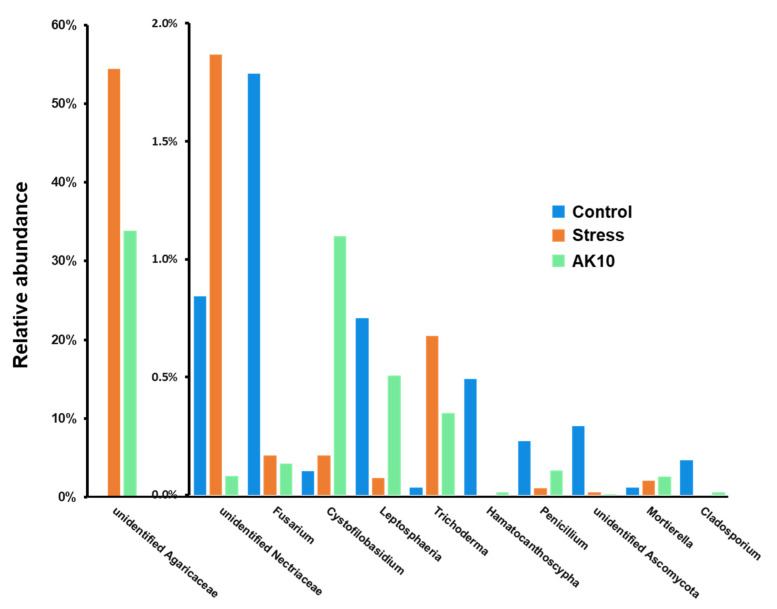
Relative abundances of fungal genera with differences across control, stressed, and AK10-treated Korean fir seedling rhizospheres. Bars denote mean values (*n* = 3) of each fungal genus relative abundance. Control represents *A. koreana* seedlings under regular watering; stressed represents seedlings exposed to drought stress; and AK10 represents seedlings both exposed to drought stress and treated with *Aureobasidium pullulans* AK10.

Regarding the structure of the rhizosphere fungal community, the bar graph in Figure 7 reveals that both drought-affected and AK10-treated groups possessed a significantly higher relative abundance of an unidentified genus within the Agaricaceae family and of *Trichoderma*. However, the AK10-treated group showed reduced levels of these genera relative to their untreated counterparts. The drought-affected seedlings had a markedly elevated relative abundance of an unidentified Nectriaceae genus, with levels in the AK10-treated group being considerably less than in the control group. On the other hand, control seedlings exhibited increased relative abundances of *Fusarium, Leptosphaeria, Hamatocanthoscypha, Penicillium,* an unidentified Ascomycota genus, and *Cladosporium*. Additionally, the AK10-treated group displayed a significantly higher abundance of *Mortierella* and *Cystofilobasidium.*

### 2.7. Correlation between Rhizosphere Bacterial and Fungal Taxa in A. koreana Seedlings

**Figure 8:**
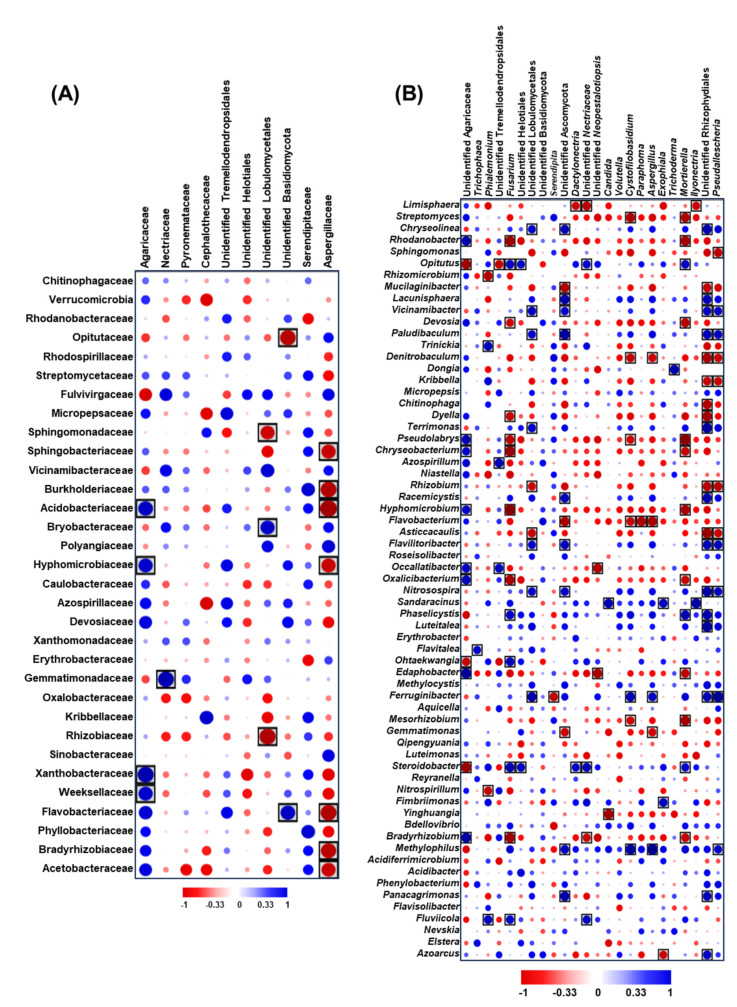
Correlogram illustrating Spearman’s rank order correlation coefficients (r) among abundant bacterial and fungal taxa within the rhizosphere micro- and myco-biome of Korean fir seedlings: (**A**) At the family level and (**B**) at the genus level. Low relative abundance taxa were excluded. Each boxed circle indicates a significant correlation: Blue for positive (*p* < 0.05) and red for negative (*p* < 0.05) and the size indicate the strength of the correlation. Unboxed circles denote non-significant correlations.

To expand our understanding of the interactions between detected bacterial and fungal taxa in the *A. koreana* rhizosphere, we conducted a correlation analysis. The correlogram in Figure 8 details Spearman’s rank order correlation coefficients (r) for the predominant bacterial and fungal taxa within the rhizosphere micro- and myco-biome of Korean fir seedlings. At the family level, as shown in Figure 8A, the Agaricaceae fungal family showed significant positive correlations with bacterial families Acidobacteriaceae, Hyphomicrobiaceae, Xanthobacteriaceae, and Weeksellaceae. The Necticeae fungal family was positively correlated with Gemmatimonadaceae, while an unidentified family within Lobulomycetales had positive correlations with Bryobacteriaceae. Another unidentified family of Basidiomycota was positively associated with Flavobacteriaceae. In terms of negative correlations, the unidentified Lobulomycetales family displayed significant negative associations with both Sphingomonadaceae and Rhizobiaceae, and an unidentified Basidiomycota family was negatively correlated with Opitutaceae. Notably, Aspergillaceae displayed negative correlations with several bacterial families: Sphingobacteria, Burkholderiaceae, Acidobacter, Hyphomicrobiaceae, Flavobacteriaceae, Bradyrhizobiaceae, and Acetobacteriaceae.

Regarding the genus-level interactions presented in Figure 8B, a notable range of significant correlations between fungal and bacterial taxa emerged, some of which potentially influence the health of *A. koreana* seedlings under drought stress. The fungal genus *Fusarium*, which may detrimentally affect plant health, exhibited negative correlations with several beneficial bacterial genera, including *Rhodanobacter*, *Devosia*, *Dyella*, *Pseudolabrys*, *Chryseobacterium*, *Hyphomicrobium*, *Oxalicibacterium*, and *Bradyrhizobium*. Similarly, *Aspergillus* displayed negative correlations with bacterial genera *Denitrobaculum*, *Flavobacterium*, and *Gemmatimonas,* but had positive associations with *Ferruginibacter* and *Methylophilus*. Another fungus, *Paraphoma*, was negatively correlated with *Flavobacterium*. In contrast, *Trichoderma*, known to potentially boost plant resilience, correlated positively with the bacterial genus *Dongia*. The complete set of correlations among the predominant taxa at the family and genus levels is provided in the correlogram of Figure 8B, highlighting the interplay between these microbial entities in the context of *A. koreana* seedlings.

## 3. Discussion

With the urgent need to protect the endangered Korean fir, it is important to note that climate change, especially drought stress, plays a significant role in the declining tree numbers. Seeking an environmentally sustainable solution to boost the tree resilience to such stress, this study isolated and identified the strain *A. pullulans* AK10 and confirmed its potential to enhance the drought tolerance in *A. koreana* seedlings. The evident efficacy of AK10 treatment in attenuating the deleterious effects of drought on *A. koreana* seedlings suggests promising applications for tree conservation. Furthermore, it highlights the need to investigate the mechanisms by which AK10 alleviates drought stress in *A. koreana*. The resilience conferred by AK10 is in line with the findings of studies that emphasize the role of microbial interactions in imparting stress tolerance to plants [23].

The isolated strain was molecularly identified as *A. pullulans*, a remarkably adaptable black yeast-like fungus. It is present in diverse environments, from soil and water to air and has the capability to colonize a broad spectrum of plant species, acting either as an epiphyte or endophyte. This range spans various plants, from apples and grapes to cucumbers and cabbages, and includes both woody tissues and leaves [24,25]. *A. pullulans* has multiple biotechnological applications, primarily stemming from its synthesis of pullulan, the biodegradable extracellular polysaccharide with usage in diverse applications [26]. Along with pullulans, this fungus produces an array of hydrolytic enzymes, enhancing its importance in environmentally sustainable applications and biotechnology [25]. These enzymes could potentially fortify soil health, facilitate nutrient cycling, or even influence plant health directly or indirectly. Furthermore, *A. pullulans* holds potential as a biocontrol agent. Previous research has highlighted various mechanisms indicating its efficacy, such as nutrient competition [27], host defense induction [28], antibiosis, and parasitism. Notably, the production of lytic enzymes, including exochitinase, endochitinase, and *β*-1,3-glucanase, is a crucial part of this efficacy [29]. Several strains have exhibited potential for biocontrol, especially against storage diseases. This antifungal activity often originates from their hydrolytic enzyme production, as the filtrates from these strain cultures maintain their antifungal properties [30]. Plant growth promotion and the production of volatile organic compounds are other mechanisms through which *A. pullulans* exerts biocontrol activity [31].

Interestingly, *A. pullulans* has been effectively vectored by bumblebees (*Bombus terrestris*) during the flowering stage of strawberries, employing the entomovectoring method. This application was shown to inhibit gray mold caused by *Botrytis cinerea* and extend the shelf life of harvested strawberries [32]. Furthermore, the antagonistic potential of *A. pullulans* against pathogens like *Aspergillus flavus* has been documented, demonstrating reduced growth both in vitro and on tomato fruits [33]. The multifaceted attributes of *A. pullulans*, encompassing antifungal capabilities, biocontrol proficiency, and plant growth promotion, are consistent with our current findings. The current research indicated that treatment with *A. pullulans* AK10 enhanced the drought stress resilience of *A. koreana*. Adding to this, the observation of modulations in the rhizosphere microbial communities further emphasizes the influential role of *A. pullulans* in ecosystem dynamics. Recent studies have also indicated the role of specific fungal species in mitigating drought stress in various trees. For instance, the ectomycorrhizal fungus, *Suillus variegatus*, has been demonstrated to enhance the growth of *Pinus tabulaeformis* trees under drought conditions. This suggests its potential application for ecological restoration in arid regions, especially within *P. tabulaeformis* forests [34]. Additionally, Zahedi et al. [35] found that native arbuscular mycorrhizal fungi can reduce the impact of drought stress on *Cercis siliquastrum* and *Prosopis cineraria* trees. In another recent study, *Trichoderma longibrachiatum* treatment was found to promote the growth of *Pinus massoniana* seedlings under drought stress. This enhancement was attributed to the regulation of physiological responses and alterations in the soil microbial community. The fungal treatment increased the abundance of beneficial microbes in alignment with our findings where *A. pullulans* AK10 ameliorated drought stress and also increased the abundance of beneficial microbiota [36].

The rhizosphere microbiome stands out as a main component within the tree holobiont, influencing its health status and stress tolerance [13]. In this context, our study investigated the effects of the proposed treatment with *A. pullulans* AK10 on the rhizosphere microbiome structure, utilizing 16S/ITS metabarcoding analysis. We observed that drought has a more pronounced impact on fungal mycobiota composition compared to bacteria, suggesting increased resilience within the bacterial community. Such drought-derived changes in the composition of the rhizosphere fungal community in *A. koreana* might contribute to seedling deterioration and rapid decline. Remarkably, treatment with *A. pullulans* AK10 appeared to counteract the significant dysbiosis, especially in fungal mycobiota composition. The weakened state of drought-stressed seedlings seems to provide an opportune environment for fungal pathogens and wood-decaying fungi to flourish [37,38]. Yet, the AK10 treatment not only attenuated these changes but also promoted the presence of certain microbial species potentially beneficial to tree health, such as the nitrogen-fixing *Azospirillum* and *Nitrospirillum* [39]. *Azospirillum*, one of the most extensively studied plant growth-promoting bacteria, has been widely recognized for its capacity to enhance plant yield [40]. This is achieved through a multifaceted approach, such as improving root development, increasing the rate of water and mineral uptake from the soil, and promoting biological nitrogen fixation, often leading to its usage as a biofertilizer. The beneficial effect of *Azospirillum* on plant root architecture is particularly noteworthy [41]. This morphological adaptation is primarily attributed to the production of phytohormones, especially indole-3-acetic acid (IAA) by the bacterium [42]. Additionally, *Azospirillum* enhances plant resilience by producing other various phytohormones, solubilizing phosphate, generating a plethora of enzymes and small-sized molecules, and enhancing membrane activity. Thus, it plays an important role in mitigating environmental stressors and competes effectively against pathogens [41].

In our study, *A. koreana* seedlings treated with AK10 displayed enhanced drought tolerance and concurrently exhibited an increased relative abundance of *Azospirillum*. This increase in *Azospirillum* and other beneficial genera abundance might contribute to the observed drought resilience in the seedlings upon *A. pullulans* treatment, highlighting the potential role of advantageous shifts in the rhizosphere microbial community. Another remarkable observation was the considerable surge in the abundance of fungal members from the Agaricaceae family in drought-affected seedlings, which almost constituted 50% of the structure. These fungi, often linked to leaf litter decomposition, seem to capitalize on the stressed state of *A. koreana* [43]. Fortunately, the AK10 treatment significantly reduced this proliferation. Furthermore, the rhizosphere of AK10-treated seedlings displayed an elevated abundance of *Cystofilobasidium*, a recognized biocontrol agent with proven efficacy against fungal diseases and has also exhibited enhanced resilience to environmental challenges, including oxidative stress [44]. Another genus, *Mortierella*, showed increased abundance in AK10-treated seedlings. This phosphate-solubilizing fungus supports crops and mycorrhizal fungi in phosphorus acquisition and also contributes to plant litter decomposition [45,46]. A previous study has also indicated that both *Cystofilobasidium* and *Mortierella* were identified in elevated concentrations in the soil around healthy plants [47].

The rhizosphere of *A. pullulans* AK10-treated seedlings exhibited a significant reduction in *Fusarium* levels, a genus known for its pathogenic members that can cause plant diseases, mainly wilting and root rot [48]. When considering all these findings, the AK10 treatment clearly exerts positive effects on the compositional structure of both fungal and bacterial communities in the rhizosphere. This could be intricately linked to the observed enhanced drought tolerance in *A. koreana*, suggesting that AK10’s influence on the rhizosphere microbial dynamics may contribute significantly to its drought resistance mechanism. This is consistent with a previous study by Han et al. [19], which highlighted distinct differences in rhizosphere microbial structures between healthy and dead Korean fir under natural conditions. Such differences emphasize the potential role of the rhizosphere in safeguarding trees and sustaining their health, even in challenging environments.

In the correlation analysis conducted between the fungal and bacterial communities within the *A. koreana* rhizosphere, we hypothesized that interactions between bacterial and fungal taxa could have significant implications for host plant health and resilience. This becomes particularly critical when plants face environmental stress, such as drought [49]. Our findings highlight distinct associations within rhizosphere bacterial and fungal communities. This aligns with the notion that microbial composition can potentially influence the broader community structure through mechanisms like co-occurrence or antagonism, as the complex interplays reported in previous studies [16,50]. The observed positive correlation between the Agaricaceae fungal family and bacterial families like Acidobacteriaceae suggests potential mutualistic relationships. Previous research indicates that co-existing fungal and bacterial taxa can synergistically augment nutrient uptake and enhance plant resistance against stresses. [51]. On the other hand, the fungal family Aspergillaceae, which includes the plant pathogenic genus *Aspergillus,* especially in dry conditions [52], showed negative correlations with bacterial taxa such as Sphingobacteria, Flavobacteriaceae, Bradyrhizobiaceae, Acidobacteriaceae, and Burkholderiaceae. Such patterns might indicate competitive or antagonistic interactions. This supports the premise that beneficial bacteria could potentially counteract the effects or growth of pathogenic fungi, thereby providing an added layer of protection to *A. koreana* seedlings under drought stress [53].

The positive association between *Trichoderma* and *Dongia* is particularly notable, given the documented bioactivities and plant growth promotion and stress tolerance enhancements of *Trichoderma* documented biocontrol potential and its activity to promote plant growth [54]. Such positive correlations are consistent with findings that certain bacterial-fungal synergies can amplify plant defenses [55]. Several other significant correlations were identified between fungal mycobiota and bacterial microbiota at the genus level. These correlations could carry multiple implications; for instance, they could help elucidate an ideal rhizosphere microbiome composition that supports trees during stress. Additionally, understanding these correlations can offer avenues for strategic manipulation to amplify the presence of key microbial members that play important roles in tree health and in controlling disease-causing microbes. An example from our data is the bacterial genus, *Flavobacteria*, which showed a negative correlation with several fungal genera, including *Paraphoma* and an unidentified Ascomycota genus. Certain fungi from these genera, especially within *Paraphoma*, are known pathogens causing diseases like crown rot and root rot and are recognized for their detrimental effects on plant growth under conditions like drought stress [56,57]. In the same line, the phytopathogenic genus *Fusarium* displayed negative correlations with several bacterial species, especially the beneficial bacteria *Bradyrhizobium* and *Chryseobacterium.* These bacterial genera are recognized for promoting plant growth, enhancing drought stress tolerance, and exhibiting antagonistic interactions with pathogenic fungi like *Fusarium* [58,59,60,61]. It is essential to emphasize that the precise nature of the observed correlations, whether they are mutualistic, simply associative, competitive, or antagonistic, requires more comprehensive studies. Such insights could offer valuable information for potential interventions, guiding the enhancement of specific microbial taxa that may influence the broader community.

## 4. Materials and Methods

### 4.1. Isolation and Culture Condition of Bioactive Agent against Drought Stress

Tree needle and stem samples were collected from pine forests in Gumi, South Korea. Following collection, the samples underwent surface sterilization by immersion in 70% ethanol for 1 min, then rinsed three times with sterile distilled water (SDW) and air-dried on sterilized filter paper. For microbial isolation, 1 g of the sterilized stems or needles was homogenized in 1 mL of SDW. The resulting homogenate was serially diluted, and 200 µL for appropriate dilution was spread onto yeast extract peptone dextrose (YPD) agar plates (Yeast extract 10 g/L, peptone 20 g/L, dextrose 20 g/L, agar 15 g/L). Plates were incubated for 2 d at 25 °C. Among the emergent bacterial and yeast colonies, a particular fungal isolate, designated as strain AK10, displayed a black-yeast-like morphology and was singled out for subsequent studies.

To maintain its viability and purity, strain AK10 was consistently cultivated on YPD medium at 25 °C. For extended storage, the AK10 cultures were preserved in YPD broth containing 30% glycerol and stored at −80 °C. When preparing the AK10 inoculum for experimental treatments, it was first cultured in YPD for 24 h at 25 °C with consistent agitation. The resulting culture was then adjusted to achieve an optical density (O.D.)_600_ = 0.8 and was used for treatment.

### 4.2. Drought Tolerance Enhancement in Abies koreana Seedlings by AK10 Treatment

To investigate the impact of AK10 treatment on drought tolerance in *A. koreana*, a seedling assay was performed. Three-year-old *A. koreana* seedlings were obtained from International Horticultural Seedlings (Seoul, Republic of Korea). These seedlings were transplanted into plastic pots (11.5 cm in diameter and 16 cm in height) containing bed soil obtained from Punong (Gyeongju, Republic of Korea). Following transplantation, the seedlings were kept in a greenhouse with conditions maintained at 25 ± 5 °C and irrigated daily. The treatment suspension was formulated as previously described. After verifying the concentration of the AK-10 culture solution, Tween-20 was added at a concentration of 250 µg/mL to the diluted culture broth. The resultant mixture was applied as a foliar spray to the seedlings on three occasions: 7 days prior, 4 days prior, and 4 days post-initiation of drought stress conditions. As a negative control, a YPD broth supplemented with Tween-20 (250 µg/mL) was used. Drought stress in the *A. koreana* seedlings was induced by discontinuing irrigation. Three weeks after inoculation, the severity of drought stress in the seedlings was assessed using a defined scale. The scale ranged from 0 to 5, where 0 = healthy seedlings with vibrant green needles; 1 = less than 20% brown wilted needles; 2 = 20–39% brown wilted needles; 3 = 40–59% brown wilted needles; 4 = 60–79% brown wilted needles along with bending of the terminal shoots; and 5 = 80–100% brown needles with the entire seedling wilting. The seedling assay was conducted twice with 3 replicates for each treatment.

### 4.3. Taxonomic Identification of AK10

The fungal strain AK10, exhibiting black-yeast-like characteristics, was identified through sequence analysis of the internal transcribed spacer (ITS) region and *β*-tubulin gene. The strain was streaked onto YPD agar plates and incubated at 25 °C. From these plates, a single colony was transferred to fresh YPD broth and incubated at 25 °C with shaking at 200 rpm. Genomic DNA was then extracted from the culture using the Wizard^®^ Genomic DNA Purification Kit (Promega, Madison, WI, USA), following the manufacturer’s guidelines. The extracted gDNA quantity and purity were ascertained via agarose gel electrophoresis and quantification with a NanoDrop™ 2000 spectrophotometer (Thermo Fisher Scientific, Waltham, MA, USA).

Subsequently, amplification of the ITS region (comprising ITS region 1, 5.8S rDNA, and ITS 2) and the *β*-tubulin (TUB) gene was performed using ITS1 and ITS4 primers, and Bt2a and Bt2b primers, respectively [62]. The PCR-amplified products were purified and sequenced. The resulting sequences were then compared to related type strains using the basic local alignment search tool (BLAST). For evolutionary analysis, phylogenetic trees were constructed employing the maximum-likelihood and neighbor-joining methods in the MEGA software (version 11) [63].

### 4.4. Metagenomic DNA Extraction

Root and adjacent soil samples were collected from three groups: Healthy seedlings that were watered regularly, seedlings under drought stress, and drought-stressed seedlings that received AK10 treatment. After discarding the closely attached soil, only the rhizosphere-enriched fractions were retained for subsequent analysis. Approximately 5 g of each harvested sample was immersed in 20 mL of SDW, followed by vigorous mixing using a vortex for 5 min. Afterward, excess water was discarded by centrifuging the mixture at 10,000× *g* for 15 min. From the resulting sediment, a 250 mg sample was used for metagenomic DNA extraction, employing the PowerSoil^®^ DNA Isolation Kit (Qiagen, Hilden, Germany; formerly Mo Bio Laboratories, Carlsbad, CA, USA), following the protocol provided by the manufacturer. The purity and concentration of the extracted metagenomic DNA from the rhizosphere were assessed through agarose gel electrophoresis and the NanoDrop2000 spectrophotometer (Thermo Fisher Scientific, Wilmington, NC, USA). Metagenomic DNA extracts meeting the desired quality and concentration criteria were preserved in a Tris-EDTA buffer and stored at −20 °C for future use.

### 4.5. Metagenomic 16S rRNA and ITS Amplification, Sequencing, Microbiome Analysis

Metagenomic analysis was performed on the hypervariable V3 and V4 regions of the 16S rRNA sequences, as well as the ITS1 and ITS2 region sequences. These regions were amplified in accordance with the protocol of the Herculase II fusion DNA polymerase Nextera XT Index Kit V2. Sequencing was conducted on the Illumina^®^ MiSeq^®^ platform at Macrogen (Seoul, Republic of Korea).

Raw paired-end sequences obtained from the sequencing were merged using the Fast Length Adjustment of Short Reads (FLASH) software (Version 1.2.11) [64]. Following this, adaptors were removed, and short reads were trimmed. The purified merged sequences were then processed and analyzed using the DADA2 package [65]. With a set cut-off value of 97%, the UCLUST algorithms were employed to cluster and annotate the purified sequences into amplicon sequence variants (ASVs). The Greengenes database was used for bacterial taxonomic assignment, while the fungal assignments were made using the UNITE database [66,67,68]. The rhizosphere microbiome analyses followed the pipeline of Quantitative Insights Into Microbial Ecology version 2 (QIIME2). This allowed for the calculation of alpha and beta diversity statistics and the taxonomic assignment of detected ASVs [69,70]. All obtained sequences have been deposited in the National Center for Biotechnology Information database as a Sequence Read Archive under the BioProject ID PRJNA1015383 for the 16S rRNA sequences and PRJNA1015390 for the ITS sequences.

### 4.6. Statistical and Data Analysis

Metabarcoding analysis of the rhizosphere from *A. koreana* seedlings, encompassing 16S rRNA and ITS regions, was conducted using QIIME2 scripts and R software (version 3.1.3). The rarefaction curves of sequenced data relative to the detected ASVs, as well as correlation analyses and correlograms, were generated with the Palaeontological Statistics software package (PAST, version 3.23) [71]. Beta diversity comparisons between samples were visualized using both weighted and unweighted UniFrac distance metrics. Heatmaps of the most abundant bacterial and fungal genera were created based on Euclidean distance measurements paired with average linkage hierarchical clustering. The drought stress index evaluation utilized a predefined scale. Data analysis was performed through the SAS (Version 9) software’s glm procedure and mean differences for the drought stress index and the alpha diversity metrics were determined using the least significant difference test at a significance level of *p* < 0.05.

## 5. Conclusions

Taken together, this study successfully isolated and identified the strain *A. pullulans* AK10, demonstrating its potential to enhance the drought tolerance of *A. koreana*. Treatment with *A. pullulans* AK10 also elevated the presence of certain microbial taxa potentially beneficial for plant growth and stress resistance. The modulations in the microbial composition by AK10 treatment could be contributing to its protective effect against drought stress in *A. koreana*. Furthermore, this research elucidates the complex interactions of rhizosphere microbes, paving the way for a more comprehensive understanding of their essential roles in this environment. Finally, the AK10 treatment represents a sustainable strategy, holding significant potential as a sustainable alternative for the conservation of this endangered tree species.

## Figures and Tables

**Table 1:** Alpha diversity metrics of the rhizosphere microbiome associated with *Abies koreana* seedlings.

Treatment	ASVs	Chao1	Shannon	Gini-Simpson
	**16S rRNA**			
**Control**	691.33 ± 81.12 ns *	692.33 ± 81.49 ns	8.54 ± 0.35 ns	0.993 ± 0.003 ns
**Stressed**	723.33 ± 117.70 ns	730.69 ± 119.61 ns	8.55 ± 0.36 ns	0.994 ± 0.002 ns
**AK10**	695.667 ± 82.98 ns	697.77 ± 83.96 ns	8.67 ± 0.26 ns	0.996 ± 0.001 ns
	**Fungal ITS**			
**Control**	246.00 ± 11.14 a **	249.24 ± 13.45 a	5.47 ± 0.02 a	0.94 ± 0.01 a
**Stressed**	107.67 ± 41.90 b	115.64 ± 40.11 b	3.23 ± 1.03 b	0.71 ± 0.13 b
**AK10**	176.33 ± 17.25 b	189.50 ± 11.87 b	4.16 ± 0.45 b	0.85 ± 0.05 b

Values represent means ± standard errors (*n* = 3). ASVs denote amplicon sequence variants; Control refers to regularly watered *A. koreana* seedlings; Stressed indicates *A. koreana* seedlings subjected to drought stress; AK10 indicates *A. koreana* seedlings subjected to drought stress and treated with *Aureobasidium pullulans* AK10. * ns, indicates no significant difference. ** Values followed by different lowercase letters represent significant differences between treatments according to the least significant difference test at *p* < 0.05.

## Data Availability

The obtained sequences were deposited in the National Center for Biotechnology Information database as a sequence read archive under BioProject ID PRJNA1015383 and PRJNA1015390.

## Supplementary Materials

The following supporting information can be downloaded at: https://www.mdpi.com/article/10.3390/plants12203653/s1, Figure S1: Rarefaction curves for 16S rRNA metagenomic sequence reads and internal transcribed spacer sequence reads from the rhizosphere of the *Abies koreana* seedlings; Figure S2: Heatmap depicting the hierarchical clustering, using Euclidean distance and average linkage, of the top 50 most abundant bacterial genera in the rhizosphere of Korean fir seedlings; Figure S3: Heatmap depicting the hierarchical clustering, using Euclidean distance and average linkage, of the top 25 most abundant fungal genera in the rhizosphere of Korean fir seedlings. Table S1: Results from the high-throughput metagenomic sequencing of the rhizosphere microbiome associated with *Abies koreana* seedlings, representing total bases, total reads, GC%, AT%, Q20%, and Q30%. Table S2: List of all detected microbes based on the 16S rRNA metabarcoding sequence analysis in the Korean fir seedling rhizosphere. Table S3: List of all detected fungi based on the ITS metabarcoding sequence analysis in the Korean fir seedling rhizosphere.
