# Supplementary material for: Aureobasidium pullulans Treatment Mitigates Drought Stress in Abies koreana via Rhizosphere Microbiome Modulation
Source: Plants (Basel). 2023 Oct 23;12(20):3653. doi: 10.3390/plants12203653 (PMC10610362; doi:10.3390/plants12203653)
Supplement: Supplementary file 1 [file plants-12-03653-s001.zip › Supplementary figures.docx]

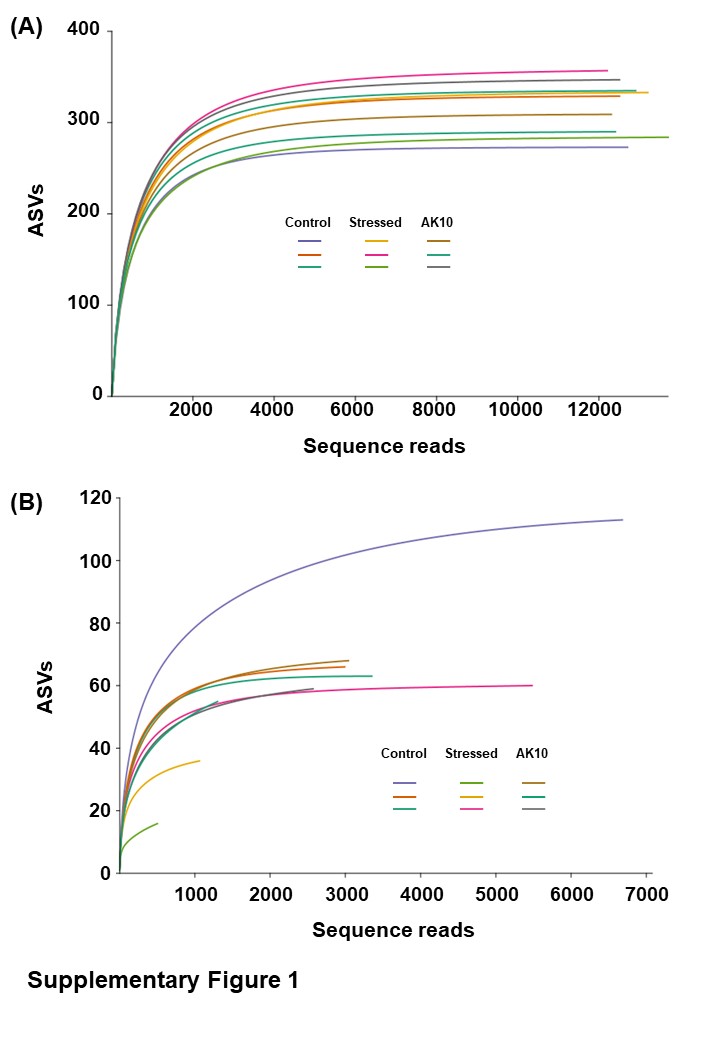


Figure S1. Rarefaction curves for 16S rRNA metagenomic sequence reads and internal transcribed spacer sequence reads from the rhizosphere of the *Abies koreana* seedlings.


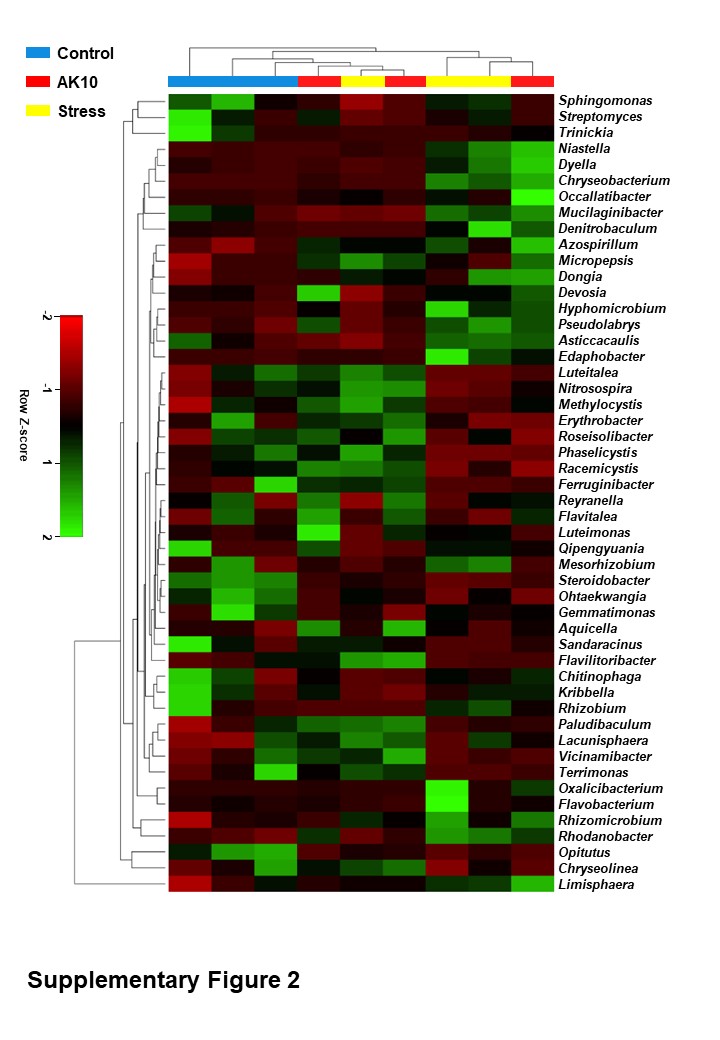


Figure S2. Heatmap depicting the hierarchical clustering, using Euclidean distance and average linkage, of the top 50 most abundant bacterial genera in the rhizosphere of Korean fir seedlings.


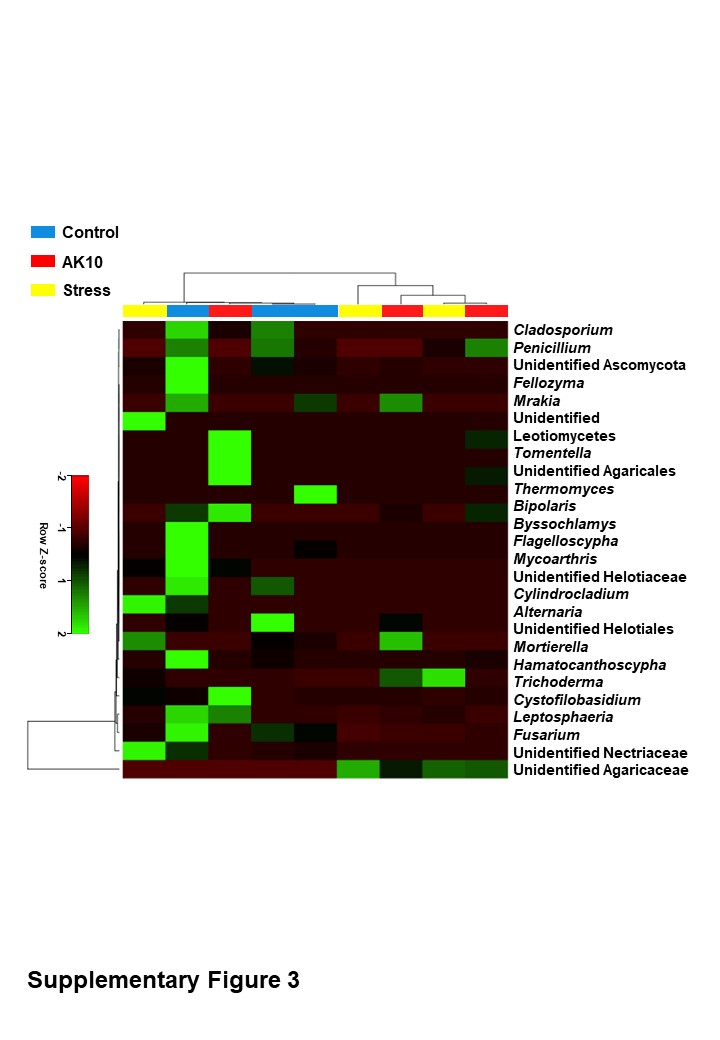


Figure S3. Heatmap depicting the hierarchical clustering, using Euclidean distance and average linkage, of the top 25 most abundant fungal genera in the rhizosphere of Korean fir seedlings.
