# Supplementary material for: Aureobasidium pullulans Treatment Mitigates Drought Stress in Abies koreana via Rhizosphere Microbiome Modulation
Source: Plants (Basel). 2023 Oct 23;12(20):3653. doi: 10.3390/plants12203653 (PMC10610362; doi:10.3390/plants12203653)
Supplement: Supplementary file 1 [file plants-12-03653-s001.zip › Table S1.doc]

**Table S1.** Results from the high-throughput metagenomic sequencing of the rhizosphere microbiome and mycobiome associated with *Abies koreana* seedlings, representing total bases, total reads, GC%, AT%, Q20% and Q30%

| **Sample** | **Total bases** | **Total reads** | **GC(%)** | **AT(%)** | **Q20(%)** | **Q30(%)** |
| --- | --- | --- | --- | --- | --- | --- |
|  | **Microbiome** | | | | | |
| Control-1 | 33,822,768 | 112,368 | 55.4 | 44.6 | 84.7 | 74.1 |
| Control-2 | 31,240,188 | 103,788 | 55.3 | 44.7 | 85.6 | 75.4 |
| Control-3 | 25,388,146 | 84,346 | 55 | 45 | 85.3 | 74.8 |
| AK10-1 | 28,528,178 | 94,778 | 55.1 | 44.9 | 86.4 | 76.8 |
| AK10-2 | 28,326,508 | 94,108 | 55.3 | 44.7 | 86.4 | 76.6 |
| AK10-3 | 27,513,808 | 91,408 | 55 | 45 | 86.2 | 76.2 |
| stress-1 | 33,333,944 | 110,744 | 55.1 | 44.9 | 85.9 | 76.1 |
| stress-2 | 29,157,870 | 96,870 | 54.8 | 45.2 | 86.6 | 76.8 |
| stress-3 | 30,945,810 | 102,810 | 55 | 45 | 87 | 77.5 |
|  | **Mycobiome** | | | | | |
| Control-1 | 41,861,274 | 139,074 | 48 | 52 | 78.5 | 67.4 |
| Control-2 | 55,650,686 | 184,886 | 46.4 | 53.6 | 75.8 | 65.6 |
| Control-3 | 70,764,498 | 235,098 | 48.5 | 51.5 | 77.2 | 65.9 |
| AK10-1 | 91,942,858 | 305,458 | 50.8 | 49.2 | 75.2 | 62.8 |
| AK10-2 | 85,676,038 | 284,638 | 48.2 | 51.8 | 77.4 | 66.5 |
| AK10-3 | 75,383,644 | 250,444 | 48.9 | 51.1 | 80.3 | 70 |
| stress-1 | 70,105,308 | 232,908 | 49.5 | 50.5 | 71.2 | 58.8 |
| stress-2 | 70,400,890 | 233,890 | 44.8 | 55.2 | 84.6 | 76 |
| stress-3 | 81,297,692 | 270,092 | 47 | 53 | 78.9 | 69.1 |

Control, *A. koreana* seedlings under regular watering; Stressed, represents seedlings exposed to drought stress; and AK10 represents seedlings both exposed to drought stress and treated with *Aureobasidium pullulans* AK10.
